# The Cellular and Humoral Immune Response to SARS-CoV-2 Messenger RNA Vaccines Is Significantly Better in Liver Transplant Patients Compared with Kidney Transplant Patients

**DOI:** 10.3390/pathogens12070910

**Published:** 2023-07-05

**Authors:** Anja Lautem, Simone Cosima Boedecker-Lips, Elisa Schneider, Stefan Runkel, Christina Feist, Hauke Lang, Julia Weinmann-Menke, Martina Koch

**Affiliations:** 1 Department of General, Visceral and Transplantation Surgery, University Medical Center Mainz, Johannes Gutenberg University, D 55131 Mainz, Germany; eschne02@students.uni-mainz.de (E.S.); hauke.lang@unimedizin-mainz.de (H.L.); martina.koch@unimedizin-mainz.de (M.K.); 2 Department of Nephrology, I. Department of Medicine, University Medical Center Mainz, Johannes Gutenberg University, D 55131 Mainz, Germany; simonecosima.boedecker-lips@unimedizin-mainz.de (S.C.B.-L.); julia.weinmann-menke@unimedizin-mainz.de (J.W.-M.); 3 Blood Transfusion Center, University Medical Center Mainz, Johannes Gutenberg University, D 55131 Mainz, Germany; stefan.runkel@unimedizin-mainz.de; 4 Department of Internal Medicine, University Medical Center Mainz, Johannes Gutenberg University, D 55131 Mainz, Germany; christina.feist@unimedizin-mainz.de

**Keywords:** SARS-CoV-2, transplantation, immunosuppression, immune response, vaccination

## Abstract

Patients after organ transplantation have impaired immune response after vaccination against the SARS-CoV-2 virus. So far, published studies have reported quite different response rates to SARS-CoV-2 vaccination, ranging from 15–79% in liver and kidney transplant recipients. Up to one year after the first vaccine dose, we analyzed the humoral and cellular immune response of 21 liver transplant (LTX) patients after vaccination with mRNA vaccines compared with 28 kidney transplant (KTX) patients. We evaluated IgG against the SARS-CoV-2 spike protein as well as SARS-CoV-2 specific T cells using an ELISpot assay that detected IFN-γ- and/or IL-2-expressing T cells. We found a cellular and/or humoral immune response in 100% of the LTX patients compared with 68% of the KTX patients. Antibody titers against the spike protein of SARS-CoV-2 were significantly higher in the LTX group, and significantly more LTX patients had detectable specific IL-2-producing T cells. The immunosuppression applied in our LTX cohort was lower compared with the KTX cohort (14% triple therapy in LTX patients vs. 79% in KTX patients). One year after the first vaccination, breakthrough infections could be detected in 41% of all organ transplant patients. None of those patients suffered from a severe course of COVID-19 disease, indicating that a partial vaccination response seemed to offer protection to immunosuppressed patients. The better immune response of LTX patients after SARS-CoV-2 vaccination might be due to less intense immunosuppressive therapy compared with KTX patients.

## 1. Introduction

Solid organ transplantation (SOT) was regarded as a risk for severe acute respiratory syndrome coronavirus-2 (SARS-CoV-2) infections as immune responses might be impaired in immunosuppressed patients. Therefore, SOT patients were prioritized in Germany for SARS-CoV-2 vaccination and received vaccination with mRNA vaccines since spring 2021 [1]. Several reports suggest a reduced antibody response in patients after SOT after two doses of a SARS-CoV-2 specific mRNA vaccine [2,3,4,5,6,7,8]. Humoral response was observed in 15–57% of KTX and in 38–89% of LTX patients [4,9,10,11,12,13,14,15] after the second vaccination. However, long-term vaccine-mediated immunity does not solely rely on a pronounced humoral response but also on the development of memory T cells [16,17]. SARS-CoV-2 antigen-specific T effector cell response in healthy individuals is well documented after vaccination [18]. The importance of the cellular immune response to vaccination has been demonstrated, as SARS-CoV-2 variants could escape the humoral but not the cellular response to vaccination [19]. However, data on T cell responses in the setting of immunosuppression suggest a reduced response, e.g., in interferon-γ (IFN-γ) release assays in SOT patients [6,20]. In those studies, 30–51% of all KTX patients examined showed cellular immune responses to a SARS-CoV-2-specific mRNA vaccine [21,22]. As has been shown in KTX patients, LTX patients might also develop a weaker immune response to SARS-CoV-2 vaccination [11,23]. Therefore, a substantial proportion of transplant recipients are likely to remain at risk for COVID-19 after two vaccine doses [7]. Moreover, as the occurrence of severe breakthrough infection after vaccination had been rarely reported in KTX patients [24], a third mRNA vaccine dose was recommended early in autumn 2021 [25]. The booster vaccination in SOT patients clearly improved SARS-CoV-2 specific antibody expression in SOT [26,27,28].

Nevertheless, the question of how long vaccine protection against breakthrough infections lasts in SOT patients and in the general population remains of interest, especially regarding the possible immune escape capacities of new virus variants. The waning of the antibody response and cellular immunity over time has already been demonstrated for the general population [29] as well as for KTX patients [30].

It is most likely that different immunosuppressive regimens have an impact on the immune response in SOT patients, and liver transplant (LTX) patients usually receive less immunosuppression compared with kidney or thoracic transplant patients. Data exist that show that LTX patients with mycophenolate mofetil (MMF) had an increased risk for humoral nonresponse after SARS-CoV-2 vaccination [10,23]. Other data have shown that vaccination failure was less likely with calcineurin inhibitor monotherapy than with other immunosuppressive regimens [11]. In our center, all KTX patients usually received induction therapy with basiliximab followed by triple immunosuppressive therapy consisting of a calcineurin inhibitor (CNI) (tacrolimus (Tac)/cyclosporine A (CyA)), an antiproliferative drug ((mycophenolic mofetil (MMF) or everolimus (Eve)), and steroids. Induction therapy is not standard in LTX in our center and most patients are treated with a dual immunosuppressive regimen.

The aim of our study was to elucidate whether the immune response to SARS-CoV-2 mRNA vaccines in LTX patients might be more favorable than the unsatisfactory results in KTX patients. In this prospective study, we present the results of the humoral and cellular immune response in kidney and liver transplant patients after two vaccine doses of BNT162b2 (Comirnaty^®^, Pfizer/BioNTech, New York, NY, USA/Mainz, Germany). The long follow-up period of one year after the first vaccination enabled us to observe the persistence of the humoral and cellular vaccination response as well as the influence of booster vaccinations, and how SOT patients coped with SARS-CoV-2 infection despite full vaccination.

## 2. Materials and Methods

### 2.1. Study Design

The study was initiated to investigate the SARS-CoV-2 specific humoral and cellular vaccination response in liver and kidney transplant recipients in comparison to a healthy control group. The study was performed in accordance with the Declaration of Helsinki of the World Medical Association. The study protocol was approved by the local ethics committee of the University Medical Center Mainz, Rhineland Palatine, Germany (Approval No. 2021-15786). All participants gave informed written consent.

**Figure 1:**
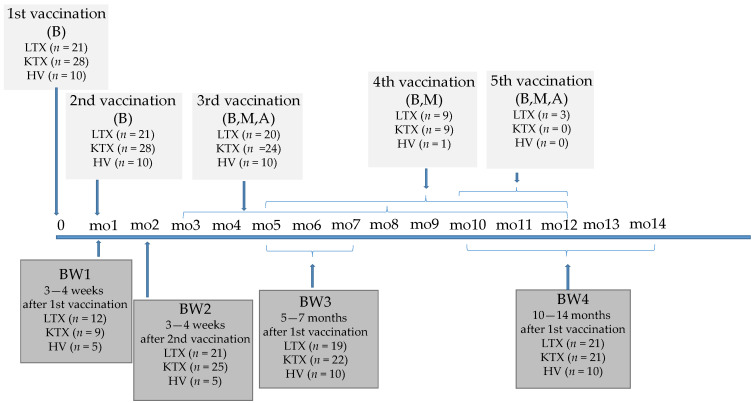
Study flow chart. Shown is the study design with the vaccination times and the measurement times of the humoral and cellular vaccination response. The number of patients examined at each time point (BW1–BW4) is indicated. It is also shown how many and in which period after the first vaccination study participants received further booster vaccinations. BW: blood withdrawal; mo: month; LTX: liver transplant patients; KTX: kidney transplant patients, HV: healthy volunteer; B: Biontech/Pfizer vaccine BNT162b2 (Comirnaty^®^); M: Moderna vaccine mRNA-1273 (Spikevax^®^); A: Astrazeneca vaccine AZD1222 (Vaxzevria^®^).

The patients were recruited from the transplant program of the University Medical Center Mainz between February 2021 and July 2022. All patients were initially vaccinated twice with BNT162b2 (Comirnaty^®^, Pfizer/BioNTech, Mainz, Germany) within an interval of four weeks. Blood samples were taken 3–4 weeks after the first (BW1) and the second (BW2) vaccination dose as well as 5–7 months (BW3) and 10–14 months (BW4) after the first vaccination. During the study, further booster vaccinations were recommended and the patients received further vaccine doses of the mRNA vaccines BNT162b (Comirnaty^®^, Pfizer/Biontech, Mainz, Germany) or mRNA-1273 (Spikevax^®^, Moderna, Cambridge, MA, USA) or AZD1222 (Vaxzevria^®^, AstraZeneca, Oxford University, Great Britain), as indicated in the study flow chart (Figure 1). In total, 3 out of 21 LTX patients received a 3rd vaccine dose before BW3 as well as 4 out of 28 KTX patients. None of the participants included had a previous SARS-CoV-2 infection, which was excluded by the determination of antibodies against the nucleocapsid protein of the SARS-CoV-2 virus.

### 2.2. Isolation of Peripheral Blood Mononuclear Cells (PBMCs)

PBMCs were freshly isolated from 20 mL heparinized blood by density gradient centrifugation using Pancoll human solution (PAN-Biotech GmbH, Aidenbach, Germany). First, the blood was diluted to half volume with phosphate-buffered saline (PBS). This mixture was pipetted gently onto the Pancoll solution using half the volume of the PBS-diluted blood solution. The gradient was immediately centrifuged for 30 min with 100× *g* without a break. The plasma was discarded and the mononuclear cell layer was transferred into a new 50 mL tube, washed twice with PBS and finally once with AIM-V medium (Gibco, Thermo Fisher Scientific, Waltham, MA, USA) for 10 min at 700× *g*, respectively. Then, cells were counted and adjusted to a concentration of 2 × 10^6^ cells/mL in AIM-V medium.

### 2.3. ELISpot Assay

To detect SARS-CoV-2-specific T cells, we performed an IFN-γ-/IL-2-ELISpot assay using the CoV-*i*Spot Interferon-γ + Interleukin-2 assay in strip format from AID Autoimmun Diagnostika GmbH (AID), Straßberg, Germany. Briefly, each strip of the 96-well plate was precoated with a mononuclear antibody against the cytokines interferon-γ (IFN-γ) and interleukin-2 (IL-2). A total of 100 µL of AIM-V medium (negative control), pokeweed mitogen (PWM) (positive control), or SARS-CoV-2 antigen solution was added in duplicate to the respective wells. The PBMC cell suspension (2 × 10^6^ cells/mL) was mixed with anti-human CD28 antibody (1:1000), and 100 µL of the prepared cell suspension was added to each well. The plate was incubated for 20–24 h at 37 °C and 5% CO_2_. After the cells had been removed and washing steps had been applied, a biotin-conjugated antibody against IL-2 together with a FITC-labeled antibody against IFN-γ was added. After two hours of incubation and further washing steps, streptavidin RED-Cy3 and anti-FITC green were added for one hour. After further washing steps, a proprietary enhancer solution was finally added and poured off after an incubation of 15 min. The plate was dried overnight and protected from light. Spot enumeration was performed with the AID iSpot Reader System (AID Autoimmun Diagnostika GmbH, Straßberg, Germany). For interpretation of the results, a stimulation index (SI) was used. Then, the mean number of spots in the antigen-containing well was divided by the mean number of spots in the negative control. Stimulated spot numbers > 7-fold higher (if the negative control was 0–1 spot) or >3-fold higher (if the negative control was 2–10 spots) than the negative control, were considered positive. Either a positive IFN-γ or a positive IL-2 result was considered indicative of a T-cell-specific immune response to vaccination.

### 2.4. Detection of SARS-CoV-2 Specific Antibodies

A qualitative chemiluminescence microparticle assay was used for detection of IgG antibodies against SARS-CoV-2 nucleocapsid protein (Architect SARS-CoV-2 IgG, Abbott GmbH, Wiesbaden, Germany) to detect previously unknown infection.

Quantitative IgG antibodies against the receptor-binding domain (RBD) of the SARS-CoV-2 spike protein were measured by chemiluminescence microparticle immunoassay (CMIA) (Architect SARS-CoV-2-IgG II Quant, Abbott GmbH, Wiesbaden, Germany) to detect vaccination response. A serological response was considered to be present at a titer >50 AU/mL, the threshold specified by the manufacturer.

### 2.5. Statistics

Data management and all statistical analyses were performed with the SPSS program (version 29.0.0.0; IBM Corp., Armonk, NY, USA). Patients’ characteristics and demographic data were displayed as mean and standard deviation (SD), if they were normally distributed and sample sizes were found to be sufficient. Non-normally distributed continuous variables were expressed as median and range, and categorial variables as number of patients and percentage. Between-group differences were analyzed by the Pearson χ^2^ test or the Fisher’s exact test, if test assumptions were not fulfilled. Normally distributed continuous data were compared using the paired *t* test, abnormally distributed data were compared by the Mann–Whitney *U* test. Differences of dependent variables were evaluated by the Wilcoxon rank sum nonparametric test. A *p* value below 0.05 was considered to indicate statistical significance.

## 3. Results

### 3.1. Patients’ Characteristics

In total, 49 SOT patients were included in our prospective study; 21 patients had a previous liver transplantation (LTX) and 28 patients had a kidney transplantation (KTX). Ten healthy volunteers (HV) served as a control group. In order to preclude prior exposure to SARS-CoV-2 virus, only participants with negative serology to SARS-CoV-2 nucleocapsid were included. All subjects received a SARS-CoV-2 vaccination with the BNT162b2 vaccine (Comirnaty^®^, BionTech/Pfizer) with an interval of 4 weeks between the first and the second dose. None of the patients reported severe side effects from the vaccination. Patients’ characteristics and demographic data are shown in Table 1.

We included 21 LTX patients, and the LTX group were compared with 28 KTX patients. The median age of the control group was comparable in both patient groups. Regarding the median, LTX patients had received transplants 4 years ago and KTX patients 3 years ago. Most LTX patients (86%) had received mono or dual maintenance immunosuppressive therapy, while most kidney patients (89%) received triple drug therapy. The ten healthy volunteers did not receive immunosuppressive medication.

None of the patients or controls acquired a SARS-CoV2 infection during the first six months of the study, up to November 2021.

### 3.2. SARS-CoV-2 Antigen-Specific Cellular Immune Response after Vaccination

**Figure 2:**
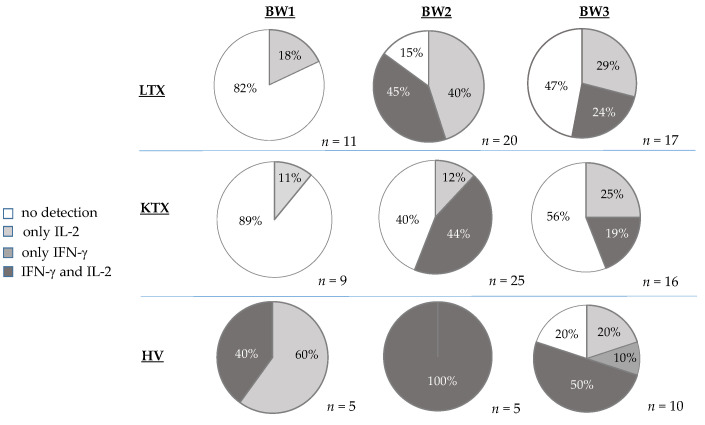
SARS-CoV-2 antigen-specific cellular response after two doses of BNT162b2 vaccine. The cellular vaccination response was measured by IL-2 and/or IFN-γ producing cells in each of the study groups four weeks after the first vaccination (BW1), four weeks after the second vaccination (BW2), and six months after the first vaccination (BW3) and is indicated as percentage of only IL-2, only IFN-γ, or IL-2- and IFN-γ-producing T cells.

To consider the SARS-CoV-2-specific immune response of LTX and KTX patients after vaccination, IFN-γ- and IL-2-producing T cells were analyzed via an ELISpot assay. The percentage of detectable IFN-γ- and IL-2-producing T cells in KTX and LTX patients, as well as in healthy controls, is shown in Figure 2.

After the first vaccination (mean 27 days (range 19 to 34 days) T-cell activity against SARS-CoV-2 was detectable but low. All of the patients were negative for INF-γ-reactive cells. Results showed that 2 out of 11 LTX samples (18%) were positive for IL-2-producing T-cells whereas 1 out of 9 KTX samples (11%) was positive for IL-2-producing T-cells. Of the healthy controls, all samples were positive for IL-2-producing T-cells and two out of five (40%) additionally for INF-γ. A markedly higher abundance of reactive T cells could be shown in healthy controls compared with LTX and KTX patients.

After the second vaccination (mean 29 days (range 19 to 62 days)), an obvious increase in the SARS-CoV-2 antigen-specific cellular response was found in LTX and KTX patients. It was found that 17/20 LTX patients (85%) were rated positive for IL-2-producing T-cells and 9 of these patients showed additional IFN-γ production (45%).

In comparison, in the KTX group only 14/25 patients (56%) had an IL-2 T-cell response. Of those patients, 11/25 (44%) had an additional IFN-γ response. After the second vaccine dose, all control samples were positive for IFN-γ- and IL-2-producing T cells.

**Figure 3:**
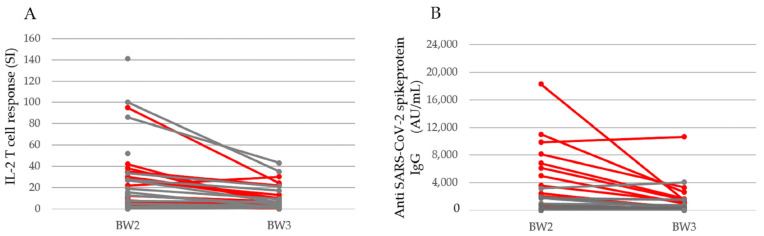
Waning of the cellular immune response in SOT patients between 4 weeks after the second vaccine dose (BW2) and 6 months following the first vaccine dose (BW3). For all solid organ transplant recipients, the stimulation index (SI) of IL-2-producing T cells (**A**) and antibody titers against the SARS-CoV-2 spike protein (AU/mL) (**B**) are indicated. The red lines represent the LTX patients, the grey lines the KTX patients.

Six months after the first vaccination (mean 177 days (range 116–206 days)), the SARS-CoV-2-specific cellular response was markedly reduced but still detectable (Figure 3). IL-2-producing cells were detectable in 9/17 LTX samples (53%), 7/16 KTX samples (44%), and 7/10 control samples (70%) but considerably reduced in all participant groups compared with BW2 (Figure 2).

Only 4/17 (24%) LTX patients and 3/16 KTX (19%) still had detectable IFN-γ-producing T cells at BW3, while these cells were found in 6/10 (60%) healthy controls.

In comparison to the KTX group, a higher number of reactive T cells could be detected in the LTX group, especially four weeks after the second vaccination.

### 3.3. Humoral Immune Response after Vaccination with BNT162b2

**Figure 4:**
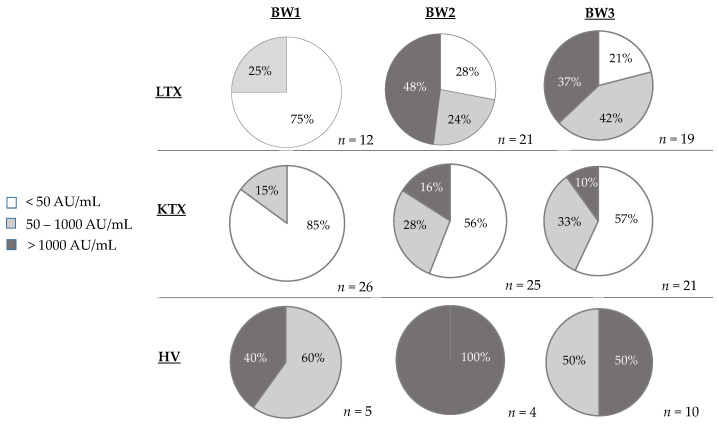
SARS-CoV-2 antigen humoral response after two doses of BNT162b2 vaccine. The humoral vaccination response was measured by IgG antibodies against the RBD of the SARS-CoV-2 spike protein four weeks after the first vaccination (BW1), four weeks after the second vaccination (BW2), and six months after the first vaccination (BW3) in each study group. Vaccination response was divided into groups of titer <50 AU/mL (negative), 50–1000 AU/mL (low/moderate), and >1000 AU/mL (high) and indicated as percentages.

After the first vaccination, most patients did not have SARS-CoV-2 IgG antibodies against the S-protein detectable above 50 AU/mL. Only 3/12 available samples from LTX patients (25%) had an IgG titer above 50 AU/mL, as well as 4/26 KTX samples (15%) (Figure 4). The titers were low, in a range from 56.3 to 112.4 AU/mL. The healthy control group had elevated IgG antibody levels against the S-protein, in a range from 410 to 1637 AU/mL (Figure 4).

After the second vaccination, 15/21 LTX patients (72%) developed IgG detectable against SARS-CoV-2 S-protein, all with titers above 100 AU/mL (median: 3603 AU/mL; range: 142–18273 AU/mL). Only 11/25 KTX samples (44%) had antibodies >50 AU/mL against SARS-CoV-2 spike protein (median 688 AU/mL; range: 64–3156 AU/mL). Antibody titers against SARS-CoV-2 S-protein were markedly higher in LTX vs. KTX patients (Figure 4). All control samples contained antibodies against the SARS-CoV-2 S-protein >1000 AU/mL (median: 5913 AU/mL; range: 2049–23717 AU/mL).

Six months after the first vaccination, antibodies against the S-protein were still detectable in 15/19 LTX patients (79%) with an AU/mL >100 (median 937 AU/mL; range: 146–10629 AU/mL). Four patients (21%) had IgG titers between 0 and 41 AU/mL. Median antibody titers of seropositive LTX patients significantly decreased from BW2 (four weeks after the second vaccination) to BW3 (six months after the first vaccination) from 3603 AU/mL to 937 AU/mL (Figure 3B). Only 9/21 available KTX patients (43%) had antibodies against the S-protein >50 AU/mL (median: 317.1 AU/mL; range 73.5–4043.7 AU/mL). In 12 patients (57%), no antibodies (<50 AU/mL) were detectable. As was shown for the LTX group, antibody titers of the KTX patients decreased from BW2 (four weeks after the second vaccination) to BW3 (six months after the first vaccination) from 688 AU/mL to 317.1 AU/mL.

In the healthy control group, all 10 samples revealed IgG against the S-protein of the SARS-CoV-2 virus (median 1122 AU/mL; range 535 and 2522 AU/mL) six months after the first vaccine dose. The antibody titers against SARS-CoV-2 were lower in KTX patients compared with LTX patients and the healthy controls. Median antibody titers also decreased in the healthy control group from BW2 to BW3, from 5913 AU/mL to 949 AU/mL.

Taking humoral and cellular immune response together, all LTX patients showed a SARS-CoV-2-specific immune response after the second vaccination (Table 2), and 60% of LTX patients had both a detectable cellular and humoral immune response. Although 25% of the LTX patients had antibody titers below 45 AU/mL, these patients showed detectable specific cellular immune responses. Furthermore, 15% of the LTX patients did not show a cellular response but had detectable antibody levels above 50 AU/mL. After KTX, only 32% had both a cellular and humoral immune response detectable. In 32% of the KTX patients, no immune response to the second vaccination was detectable. All healthy controls tested showed humoral and cellular response to SARS-CoV-2 four weeks after the second vaccination.

In most patients with an immune response four weeks after second vaccination (BW2), IL-2-secreting cells and/or S-protein-specific antibodies could still be detected after 6 months (BW3), although in a lower manner. The decrease in the immune response was most striking concerning the LTX patient group (Figure 3).

### 3.4. Immunosuppression and Immune Response

In the LTX group, all five patients with no antibody response four weeks after the second vaccination were on dual immunosuppression (Tac/MMF (*n* = 2), CyA/MMF (*n* = 1), Eve/MMF (*n* = 1)); one patient received mono immunosuppression with Tac. However, all those patients who did not show humoral response had detectable levels of SARS-CoV-2-specific T cells. Nevertheless, four weeks after the second vaccination, all LTX patients showed at least either humoral or cellular immune response independent of the immunosuppressive regimen.

KTX patients were treated more frequently with a triple immunosuppression regimen than LTX patients. All eight KTX patients without T- or B-cell response four weeks after the second vaccination received triple immunosuppression (Tac/Eve/steroids (*n* = 4), Tac/MMF/steroids (*n* = 3), and Eve/MMF/steroids (*n* = 1)).

### 3.5. SARS-CoV-2 Specific Immune Response One Year after the First Vaccine Dose

As many patients showed a rapid decline of humoral as well as the cellular immune response six months after the first vaccine dose (Figure 3), further booster vaccinations were recommended. Regarding the LTX patients, 20 out of 21 patients received a 3rd dose (95%), 9 a 4th dose (43%), and 3 a 5th dose (14%) of an mRNA vaccine or, in a single case, of the adenoviral vaccine AZD1222 (Vaxzevria^®^, AstraZeneca, Oxford University) at BW4 (1 year after the 1st vaccine dose) (Figure 1). In total, 24 out of 28 KTX patients received a 3rd dose (86%) and 9 a 4th dose (32%) of an mRNA vaccine. None of the patients reported severe side effects from the vaccination.

The humoral and cellular Immune responses were examined one year after the first vaccine dose (BW4). Moreover, one year after the first vaccination, all transplanted patients and healthy volunteers filled out a questionnaire in which they were asked whether they had experienced a proven SARS-CoV-2 virus infection. Due to anti-spike protein antibody titers beyond 40,000 AU/mL as well as anti-SARS-CoV-2 nucleocapsid-specific IgG, IgM, or IgG/IgM/IgA titers, further patients and healthy volunteers were detected that had had an asymptomatic infection. All patients and healthy volunteers developed a significant SARS-CoV-2 specific antibody response after infection.

Altogether, 7 out of 21 LTX patients (33%), 9 out of 28 KTX patients (32%), and 3 out of 10 healthy volunteers (30%) had a SARS-CoV-2 infection despite full vaccination. Five out of seven infected LTX patients and one out of ten healthy volunteers were asymptomatic and did not realize that they had a SARS-CoV-2 infection. All nine infected KTX patients showed mild symptoms of COVID-19 disease. In summary, 16 out of 39 transplant patients were infected with SARS-CoV-2 virus (41%) despite full vaccination.

Although, all infected patients were vaccinated three times (*n* = 10), four times (*n* = 5) or five times (*n* = 1), not all of them developed a full immune response (SARS-CoV-2 specific T cells and antibodies) after vaccination and before infection. Namely, 3/7 patients (43%) in the LTX group and 1/9 in the KTX group (11%) did not show SARS-CoV-2 specific T cells, 1/7 in the LTX group (14%) and 2/9 patients in the KTX group (22%) did not develop neither T cells or antibodies. One patient in the KTX group did not develop antibodies before infection. All infected healthy volunteers had a T-cell response as well as antibodies before infection.

Nevertheless, all transplant patients and all healthy volunteers had mild or asymptomatic courses of COVID-19 disease. None of the patients were hospitalized and none needed ventilation with oxygen or treatment with extracorporeal membrane oxygenation (ECMO).

## 4. Discussion

In our study, we analyzed not only the humoral but also the cellular immune response of LTX and KTX patients in comparison to a healthy control group. Therefore, we used an ELISpot assay displaying not only IFN-γ but also IL-2-producing SARS-CoV-2-specific T cells. We observed a lower humoral and cellular response in KTX compared with LTX patients, which might be due to a higher degree of immunosuppression in KTX patients. After the first vaccine dose, we observed a poor immune response in SOT patients in terms of the antibody (LTX 25%; KTX 15%) as well as the cellular response (LTX 18%; KTX 11%). The situation changed after the second vaccination. Then, we detected an IL-2 response in 85% of the LTX patients and in 56% of our KTX patients. An IFN-γ response could only be detected in 45% of the LTX and in 44% of the KTX patients. It is of interest that IL-2-producing T cells were more common than IFN-γ-producing cells in our patients after vaccination, since Fava et al. [31] showed that a higher number of IL-2-producing T cells after SARS-CoV-2 infection correlates with a better clinical outcome. Most of the IFN-γ-producing T cells in our patients also secreted IL-2, but we also detected several IL-2-producing T cells without IFN-γ production and we could not detect T cells that secreted IFN-γ but no IL-2. The detected T cells might contribute to a long-lasting immune response.

Several studies on the immune response to SARS-CoV-2 vaccination after solid organ transplantation are available showing a deprived humoral and cellular immune response. Our data are in accordance with data from Boyarsky et al. [32] and Benotmane et al. [33] who have already shown that after the first vaccination no adequate response can be expected in immunosuppressed patients. Most published studies predominantly include kidney transplant patients [6,7,8,20,34,35], but also thoracic transplant patients [5] and liver transplant patients [4,36]. Most data focus on the examination of the antibody response to SARS-CoV-2 vaccination [3,4,9,10]. Although it has been suggested that T-cell immunity plays an important role after SARS-CoV-2 infection [17,19], the investigation of the cellular immune response is rather scarce and is often limited to the measurement of IFN-γ-producing T cells [11]. Our results demonstrate a much better outcome in terms of SARS-CoV-2 spike protein-specific T-cell response than other data that solely measured IFN-γ-producing T cells to characterize the immune response to vaccination [11].

Furthermore, we were able to demonstrate a significant difference in antibody response between LTX and KTX patients after the second vaccination. While the majority of the LTX patients (72%) had antibody titers after the second vaccination, only 44% of the KTX patients had an antibody response >50 AU/mL against the SARS-CoV-2 spike protein, which is in accordance with previously published data [6,32]. Also, significantly higher antibody levels were found in our study for LTX patients compared with KTX patients. We would like to point out that almost three quarters of the LTX patients developed antibody titers above 1000 AU/mL. Furthermore, 56% of all KTX patients did not show seroconversion, but 24% of those patients without antibody response had detectable specific T cells. This phenomenon was even more pronounced in the LTX group; 38% of the LTX patients did not show antibodies after the second vaccination but all of them revealed specific T cells. All the LTX patients in this study revealed some kind of immune response; all of them showed at least antibody or T-cell response if not both. The immune response of our KTX cohort was lower, as 32% did not show any response to the second vaccination. These findings give an optimistic outlook on the outcome of SARS-CoV-2 vaccination, especially for LTX patients. Our data reveal that vaccinated transplant patients under immunosuppression might have more protection against severe courses of COVID-19 disease than they were initially supposed to have. This is in accordance with the clinical courses of our patients, showing no severe COVID-19 disease in transplant patients.

In our study, because of the long-term follow-up of one year after the first vaccine dose, we were able to regard the persistence of the immune response over a long time period. Moreover, we could observe the clinical outcomes after breakthrough infections. Six months after the first vaccination, we detected reduced humoral response and less T-cell response compared with the second blood withdrawal four weeks after the second vaccination. Our findings are in accordance with the studies by Bertrand et al. [30] who also showed the waning of humoral and cellular immune response at a later time point, namely, six months after the third dose of BNT162b2 vaccine. A study by Goldberg et al. [29] also indicated that immunity against SARS-CoV-2 waned a few months after receipt of the second vaccine dose. Due to the reduced cellular and humoral immune response detected after six months both in patients and healthy volunteers, booster vaccinations were recommended as early as three months after the second vaccination [37]. Hence, our study patients received quite different booster vaccination regimens concerning the number of booster vaccinations and the applied type of mRNA vaccine, leading to cross-vaccination in many cases. Because of the relatively small patient numbers in our cohort, we could not draw any conclusion concerning the influence of booster or cross-vaccinations on the SARS-CoV-2 specific immune response. However, it was evident that patients that did not develop any immune response after the second vaccination generally did not even show seroconversion or specific T cells after the third vaccination, independently of the applied booster vaccine. Data from case series support this and seem to indicate that there might still be no better or only slightly better seroconversion after a fourth compared to a third vaccination [38,39].

Nevertheless, we assume that the high rate of IL-2-producing cells detectable in our patients after the second vaccination (LTX 85%; KTX 56%) and even after six months (LTX 53%; KTX 44%) might be good news, especially for LTX patients, since the immune response to vaccination might be underestimated when looking only at antibody expression or IFN-γ-producing T cells.

The different immune response of LTX and KTX patients is most likely to be explained by the difference in immunosuppressive regimens. As the liver shows higher immune tolerance after transplantation, a lower dosage of immunosuppression is needed compared with other transplanted organs such as the kidney. It has been shown that MMF and triple drug immunosuppression are risk factors for an inferior immune response to SARS-CoV-2 vaccination [4]. While 25 out of 28 KTX patients received standard triple drug therapy consisting of CNI, steroids, and an anti-proliferative drug, only 3 of 21 LTX patients received triple drug therapy. Due to the low numbers of patients and several different drug combinations included, we cannot draw conclusions about the effects of specific drugs. The result that four of six patients vaccinated within the first year after kidney transplantation developed neither T-cell nor humoral immune response leads to the assumption that the total burden of immunosuppression negatively influences the immune response. This is an expected result, since other vaccines also show an inferior immune response within the first year post transplantation [40]. The different immunosuppressive regimen together with the later time point of blood sampling in the study by Rabinowich et al. [4] (mean 31 days after the second vaccination compared with 10–20 in the study by Rabinowich et al.) might explain the clearly higher immune reactivity in our LTX patients. The difference in the response rates to SARS-CoV-2 vaccination in addition to the immunosuppressive regimen was also reported to be dependent on age or time after transplantation [4,7,9]. In comparison to other studies [4,9,13] where the mean age of transplant patients was >60 years, our cohort was younger, as the mean age of our LTX patients was 57 years and that of the KTX patients was 50 years.

Although it is not clear whether the T-cell and/or antibody response correlates with protection against severe SARS-CoV-2 infection, our data provide evidence that the effect of two doses of SARS-CoV-2 mRNA vaccine BNT163b2 after liver transplantation is more effective than vaccination after kidney transplantation. However, until now, no antibody or specific T-cell threshold has been established for protective immunity. The high amount of IL-2 as well as IL-2/IFN-γ-producing T cells detected in LTX patients may lead to the assumption that these patients might have adequate long-term protection against severe SARS-CoV-2 infections. Although we included only a small number of patients in our study, it is encouraging that none of the studies’ patients developed a SARS-CoV-2 infection in the first six months after initial vaccination. It remains a matter of debate whether this was due to the vaccination alone or also to the high hygienic standards applied to transplant patients. According to the current knowledge, it should be remembered that the aim of the vaccine is not that the patient does not acquire the infection but that it prevents patients from progressing and developing respiratory failure.

In our cohort, in 16 of 39 SOT patients a breakthrough infection was determined at the fourth blood withdrawal one year after the first vaccination (BW4). Seven patients with breakthrough infections did not show a cellular immune response before infection, four did not have detectable antibodies, and three developed neither T cells nor antibodies six months after the first vaccination independent of further booster vaccinations. Thus, 86% of the LTX and 89% of the KTX patients with breakthrough infection showed either no immune response or an incomplete immune response with a lack of antibody response in the presence of T cells or, vice versa, with a lack of T cells in the presence of seroconversion. Interestingly, the breakthrough infections with SARS-CoV-2 virus occurred in LTX and KTX study patients between six (BW3) and twelve months (BW4) after the first vaccination despite further booster vaccinations. This could most probably be explained by the upcoming new virus variants BA.4 and BA.5 in this period that revealed higher immune escape capability and the minor immune response in transplant patients. Nevertheless, none of these patients with breakthrough infections suffered from severe COVID-19 disease. Beyond that, five of seven LTX patients had an unknown and asymptomatic infection. This might be due to infection with less aggressive virus variants, but it could also provide evidence that even immunosuppressed patients were sufficiently protected from at least severe SARS-CoV-2 infection even though they might develop an incomplete immune response to vaccination.

The limitations of our work include the rather small cohort of only 39 LTX and KTX patients, but the rather long examination period of one year post vaccination enabled us to examine the persistence of the immune response over a long follow-up time in transplant patients and to observe how immunosuppressed patients cope with SARS-CoV-2 breakthrough infections.

## 5. Conclusions

LTX patients developed a better immune response after vaccination with BNT162b2 than KTX patients, which might be due to different immunosuppressive therapy. LTX and KTX patients seemed to be sufficiently protected against severe COVID-19 by vaccination with mRNA vaccines, as none of the patients developed respiratory failure after SARS-CoV-2 infection.

## Figures and Tables

**Table 1:** Patients’ characteristics and demographic data.

	LTX (*n* = 21)	KTX (*n* = 28)	Control (*n* = 10)
Gender (male/female)	10/11	19/9 *	4/6
Age at first vaccination, years median (IQR 25–75)	59 (53–67)	54 (37–61) *	54 (43–58)
Years since transplantation median (IQR 25–75)	4 (1.5–11)	3 (1–11)	
Immunosuppression regimen, *n* (%)			
triple therapy	3 (14.0)	25 (89.0)	
Tac ^1^, MMF ^2^, prednisone	2	17	
CyA ^3^, MMF, prednisone	0	1	
Tac, mTORinhibitor, prednisone	1	6	
mTORinhibitor, MMF, prednisone	0	1	
dual therapy	9 (43.0)	3 (11.0)	
Tac, MMF	3	1	
Tac, mTOR inhibitor	4	0	
CyA, MMF	1	0	
CyA, prednisone	0	1	
mTOR inhibitor, MMF	1	0	
mTOR inhibitor, prednisone	0	1	
mono therapy	9 (43.0)	0 (0.0)	
Tac	7	0	
CyA	2	0	

^1^ Tac: tacrolimus; ^2^ MMF: mycophenolic mofetil; ^3^ CyA: cyclosporine A. * age and gender differences between LTX and KTX group were not statistically different.

**Table 2:** Measurements of SARS-CoV-2 antigen-specific humoral and cellular response. Shown is the humoral and cellular vaccination response, measured by anti SARS-CoV-2 specific antibodies against the spike protein and by IL-2- and/or IFN-γ-producing cells four weeks after the first vaccination (BW1), four weeks after the second vaccination (BW2), and six months after the first vaccination (BW3).

		BW1	BW2	BW3
LTX	Neither T-cell nor IgG response, *n* (%)	6/11 (55%)	0/20 (0%)	2/18 (11%)
	T-cell response only, *n* (%)	2/11 (18%)	5/20 (25%)	1/18 (6%)
	IgG response only, *n* (%)	3/11 (27%)	3/20 (15%)	7/18 (39%)
	IgG and T-cell response, *n* (%)	0/11 (0%)	12/20 (60%)	8/18 (44%)
KTX	Neither T-cell nor IgG response, *n* (%)	8/9 (89%)	8/25 (32%)	6/16 (37%)
	T-cell response only, *n* (%)	1/9 (11%)	6/25 (24%)	2/16 (13%)
	IgG response only, *n* (%)	0/9 (0%)	3/25 (12%)	3/16 (19%)
	IgG and T-cell response, *n* (%)	0/9 (0%)	8/25 (32%)	5/16 (31%)
HV	Neither T-cell nor IgG response, *n* (%)	0/5 (0%)	0/5 (0%)	0/10 (0%)
	T-cell response only, *n* (%)	0/5 (0%)	0/5 (0%)	0/5 (0%)
	IgG response only, *n* (%)	0/5 (0%)	0/5 (0%)	2/10 (20%)
	IgG and T-cell response, *n* (%)	5/5 (100%)	5/5 (100%)	8/10 (80%)

## Data Availability

All data are provided by the corresponding author.
